# Variations in Triterpenoid Deposition in Cuticular Waxes during Development and Maturation of Selected Fruits of Rosaceae Family

**DOI:** 10.3390/ijms21249762

**Published:** 2020-12-21

**Authors:** Soyol Dashbaldan, Cezary Pączkowski, Anna Szakiel

**Affiliations:** 1 Department of Plant Biochemistry, Institute of Biochemistry, Faculty of Biology, University of Warsaw, 1 Miecznikowa Street, 02-096 Warsaw, Poland; dsoyloo89@gmail.com (S.D.); myhacp@biol.uw.edu.pl (C.P.); 2 Mongolian University of Science and Technology, School of Industrial Technology, 8nd khoroo, Baga toiruu 34, Sukhbaatar district, Ulaanbaatar 14191, Mongolia

**Keywords:** cuticular wax, esterification, fruit development, fruit ripening, hydroxylation, Rosaceae, steroids, triterpenoids, ursolic acid

## Abstract

The process of fruit ripening involves many chemical changes occurring not only in the mesocarp but also in the epicarp, including changes in the triterpenoid content of fruit cuticular waxes that can modify the susceptibility to pathogens and mechanical properties of the fruit surface. The aim of the study was the determination of the ripening-related changes in the triterpenoid content of fruit cuticular waxes of three plant species from the Rosaceae family, including rugosa rose (*Rosa rugosa*), black chokeberry (*Aronia melanocarpa* var. “Galicjanka”) and apple (*Malus domestica* var. “Antonovka”). The triterpenoid and steroid content in chloroform-soluble cuticular waxes was determined by a GC-MS/FID method at four different phenological stages. The profile of identified compounds was rather similar in selected fruit samples with triterpenoids with ursane-, oleanane- and lupane-type carbon skeletons, prevalence of ursolic acid and the composition of steroids. Increasing accumulation of triterpenoids and steroids, as well as the progressive enrichment of the composition of these compounds in cuticular wax during fruit development, was observed. The changes in triterpenoid content resulted from modifications of metabolic pathways, particularly hydroxylation and esterification, that can alter interactions with complementary functional groups of aliphatic constituents and lead to important changes in fruit surface quality.

## 1. Introduction

Fruit development and ripening are dynamic processes that involve a complex series of morphological, molecular and biochemical changes. In fleshy fruits, these changes comprise modification of cellular compartments, loss of cell wall structure causing tissue softening, transformation of pectins into water-soluble forms, breakdown of storage polysaccharides and degradation of acids, as well as accumulation of pigments. Fruit development may essentially be regarded as a consequence of the interplay between various processes such as cell division, cell expansion and the gradual establishment of the composition of primary and secondary (specialized) metabolites in fruit tissues [1,2,3].

A fruit’s surface is covered with a hydrophobic layer called a cuticle. The cuticle provides mechanical support for fruit integrity and prevention against excessive fruit softening, but it is also crucial for protection against desiccation, as it minimizes water loss, and conveys resistance to pathogens [4]. The minimization of water loss and protection against both biotic and abiotic factors are the major factors influencing fruit development and post-harvest quality. Thus, the fruit cuticle affects important quality traits, such as fruit appearance, texture, firmness, mechanical toughness, susceptibility to microbial infections, disorders and surface cracking. In addition, controlling gas and water exchange with the environment, filtering potentially damaging UV light and limiting invasion by pathogens are strong determinants of fruit quality and storability characteristics [5].

Several types of crops have been well characterized in terms of their fruit cuticle. Different cultivars, varieties, related wild species or mutants display a significant degree of variability in cuticle thickness, mass and density, chemical composition and biophysical properties [6,7,8]. In general terms, the cuticles of fruits are composed of the same main classes of constituents as are found in vegetative organs (such as cutin and waxes), and their distribution in fruit surface layers is similar. However, usually the fruit cuticle is much thicker in fruit than in leaves and it contains practically no stomata. In addition, the chemical composition of cuticular wax differs significantly from that of vegetative organs of the same plant. Cuticular composition and arrangement undergo very dynamic changes, including remodeling and deep restructuring, during fruit ontogeny, development and ripening. Enhanced biosynthesis, modification of structurally significant polymers and incorporation of new material must be effectively coordinated particularly with fast expansion of the fruit surface during the phase of intensive growth [4,5]. Cuticular wax deposition starts early in fruit development; nevertheless, the pattern of wax load varies markedly among plant species. In many fruits, cuticular wax load increases during fruit development, leading to a thick cuticle at maturity, while in other species, the initial high cuticular wax deposition rate is followed by its significant reduction at later stages. It has been often observed that cuticle deposition ceases at the phase of the transition between fruit growth and ripening; however, this is not a universal pattern in all fruits [4,8].

Triterpenoids and steroids are isoprenoids produced by folding and cyclization of their common C30 precursor, the long-chain hydrocarbon squalene. They are characteristic constituents of plant cuticular waxes and particularly abundant in some fruits, while occurring only in trace amounts in others [7,9]. Occurrence of triterpenoids in fruit cuticular waxes is valuable for potential consumers since these compounds have numerous biological activities such as anti-inflammatory, antimicrobial, antiviral, cardio- and hepatoprotective, as well as potentially anticancer properties [10]. In turn, the contribution of triterpenoids to various functions of the fruit cuticle is still a subject of research and speculations. It is commonly accepted that the composition rather than the quantity of cuticular waxes is a crucial determinant of water permeability of the cuticle, and high levels of cyclic triterpenoids and a low concentration of alkanes correlate with an increased water permeance [11]. The antifungal activity of cuticular waxes of some fruits seems to be associated with triterpenoids together with some n-alkanes [12]. In addition, triterpenoids seem to enhance the mechanical strength of the fruit cuticle by functioning as nano-fillers [13]. Triterpenoids can also be responsible for photoprotection of colorless fruits in which other UV protectants such as flavonoids, anthocyanins, carotenoids or betalains are not present [14].

The pattern of changes in triterpenoid content during fruit ripening can be significant for susceptibility to pathogens as well as the mechanical properties of the fruit surface [4,8]. Nevertheless, the changes occurring in triterpenoid accumulation in cuticular waxes during fruit development are not uniform in various plant species. For example, an initially high level of triterpenoids followed by a gradual decrease in their content until full ripening was observed in the case of grapes (*Vitis vinifera*) [15] and sweet cherry (*Prunus avium*) fruit [16], as well as in some fruits from plants of the Ericaceae family, such as lingonberry (*Vaccinium vitis-idaea*) and strawberry tree (*Arbutus unedo*) [9]. In contrast, the accumulation of triterpenoids rather constantly increased during fruit development of olive (*Olea europaea*), although the profile of these compounds was altered according to the different phases in the process of maturation [17].

The understanding of the relationships between fruit cuticular composition, ripening-associated changes and the resulting fruit traits, including post-harvest quality, is still preliminary. Meanwhile, the predicted implications of cuticular composition and developmental patterns for such features as mechanical resistance and fruit susceptibility to microbial infections could have a great impact on cultivation and post-harvest strategies, such as reducing the utilization of pesticides and artificial fruit coatings. Although the application of external coatings on the fruit surface has become a popular post-harvest treatment due to its potential for extending the fruit shelf life [18], consumers prefer the natural fruit and have begun to avoid artificial waxes. Considering all these issues, the diversity and dynamic nature of fruit cuticle composition and architecture during fruit development deserve to be further explored.

The aim of the present study was the determination of the development and ripening-associated changes in triterpenoid profile of fruit cuticular waxes in three plant species from the Rosaceae family bearing edible accessory fruits, including rugosa rose (*Rosa rugosa* Thunb.), black chokeberry (*Aronia melanocarpa* (Michx.) Elliot variety “Galicjanka”) and apple (*Malus domestica* Borkh. variety “Antonovka”). The triterpenoid and steroid content in chloroform-soluble cuticular waxes was determined by a GC-MS (gas chromatography–mass spectrometry) method at four different phenological stages: very young fruits collected approximately one week after flower pollination, young fruit collected during the growth phase, fruit at the onset of ripening and, lastly, mature fruit. The obtained results complement existing data on the diversity of triterpenoid content in fruit, as well as the pattern of changes in cuticular wax composition during fruit development.

## 2. Results

### 2.1. Rugosa Rose (Rosa Rugosa Thunb.)

Chloroform extracts of cuticular waxes obtained from rugosa rose pseudo-fruits (accessory fruits), referred to as “hips”, at four consecutive stages of development were fractionated by preparative TLC (thin-layer chromatography) as described in Section 4.3. Obtained fractions were individually subjected to GC-MS/FID analysis (Section 4.6.).

**Figure 1:**
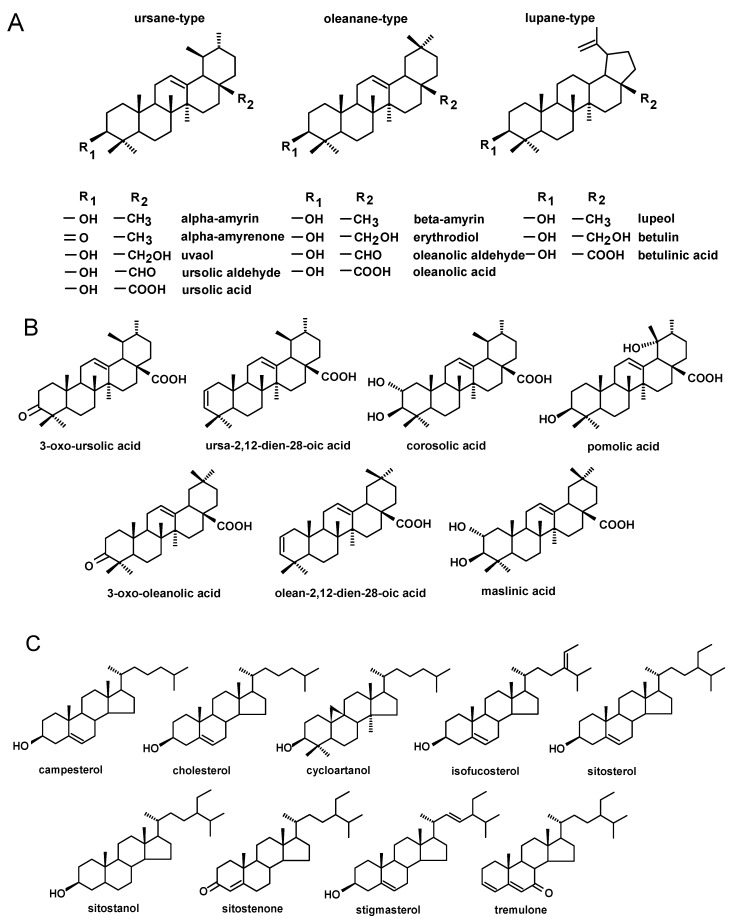
The structures of alcohols, ketones, aldehydes and acids of oleanane-, ursane- and lupane-type skeletons (**A**), derivatives of oleanolic and ursolic acids (**B**) and steroids (**C**) identified in cuticular waxes of fruit analyzed in this study.

The triterpenoid profile of rugosa rose hip cuticular wax consisted of pentacyclic compounds of three basic carbon skeletons: oleanane-, ursane- and lupane-type. Oleanane- and ursane-type skeletons consist of five rings, each containing six carbon atoms, whereas the lupane-type skeleton differs in the structure of the last ring, which is five-membered with an isopropylidene group attached (Figure 1A). The composition of the fraction of neutral triterpenoids, including alcohols, ketones and aldehydes, was rather simple and consisted only of monohydroxy alcohols. These included α-amyrin (urs-12-en-3β-ol), β-amyrin (olean-12-en-3β-ol), lupeol (lup-20(29)-en-3β-ol) and one ketone, α-amyrenone (urs-12-en-3-one), as well as oleanolic aldehyde (3β-hydroxy-olean- 12-en-28-al) and ursolic aldehyde (3β-hydroxy-urs-12-en-28-al). No dihydroxy alcohols were detected in cuticular waxes of rugosa rose hip at any stage of development. Two of these compounds, α-amyrin and lupeol, formed one common peak, which had been observed in our previous studies [9,19]. The identification of these compounds in the mixture was confirmed by GC-MS analysis of their authentic standards and examined separately or combined [19,20].

**Figure 2:**
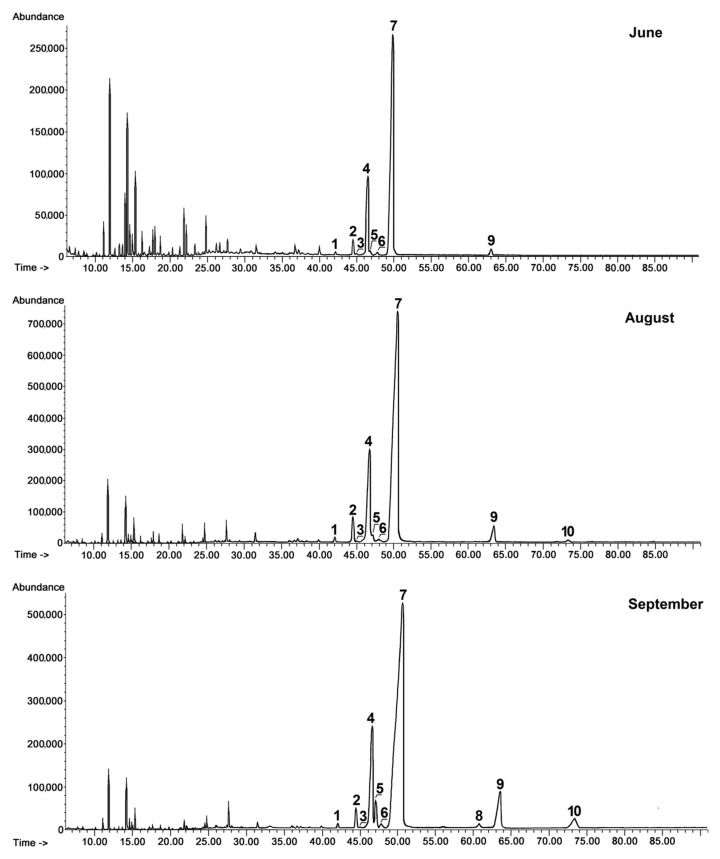
GC-FID chromatograms of the fraction of triterpenoid acids obtained from chloroform extracts of cuticular waxes of rugosa rose fruit harvested in three developmental stages (young rose hips in June, unripen full-size hips in August and ripened hips in September). Peaks of identified acids are numbered according to growing retention time: 1—olean-2,12-dien-28-oic acid, 2—ursa-2,12-dien-28-oic acid, 3—3-oxo-olean-12-en-28-oic acid, 4—oleanolic acid, 5—betulinic acid, 6—3-oxo-urs-12-en-28-oic acid, 7—ursolic acid, 8—maslinic acid, 9—corosolic acid, 10—pomolic acid (analyzed in the form of methyl esters). Peaks not numbered were associated with aliphatic compounds (e.g., methylated fatty acids).

In turn, the fraction of triterpenoid acids had a very complex composition, consisting of oleanolic acid (3β-hydroxy-olean-12-en-28-oic acid) and ursolic acid (3β-hydroxy-urs-12-en-28-oic acid), as well as betulinic acid (3β-hydroxy-lup-20(29)-en-28-oic acid), accompanied by various derivatives of oleanolic and ursolic acids including 3-oxo-analogs (3-oxo-olean-12-en-28-oic acid and 3-oxo-urs-12-en-28-oic acid), analogs with an additional double bond in position 2 (olean-2,12-dien-28-oic acid and ursa-2,12-dien-28-oic acid), 2,3-dihydroxy analogs (2α,3β- dihydroxy-olean-12-en-28-oic and 2α,3β-dihydroxy-urs-12-en-28-oic acids, i.e., maslinic and corosolic acids) and 3,19-dihydroxy-urs-12-en-28-oi acid (pomolic acid) (Figure 1B). However, maslinic and pomolic acids did not occur in the wax of young fruit and these appeared only at the later stages of development. The characteristic pattern of changes in the content of triterpenoid acids in rugosa rose hip cuticular wax can be observed in chromatograms shown in Figure 2.

The profile of steroids in the cuticular wax of rugosa rose hip was composed of several typical sterols characteristic of higher plants, including campesterol (24*R*-ergost-5-en-3β-ol), isofucosterol (24Z-stigmasta-5,24(28)-dien-3β-ol, synonym: Δ5-avenasterol), sitosterol (stigmast-5-en-3β-ol) and stigmasterol (22*E*-stigmasta-5,22-dien-3β-ol). In addition, a small cholesterol (cholest-5-en-3β-ol) peak was also detected. Among steroidal derivatives, only one ketone, tremulone (stigmasta-3,5-dien-7-one), was identified (Figure 1C). The fraction of esters, analyzed after hydrolysis as described in Section 4.5, contained mainly esters of oleanolic and ursolic acids such as α-amyrin/lupeol and sitosterol.

The quantification of individual compounds, prepared using an external standard method based on the FID (flame ionization detector) signal as described in Section 4.6, is presented in Table 1. Due to the coelution of α-amyrin and lupeol, forming one common peak, their amounts were quantified together as described in previous studies [9]. The total content of steroids and triterpenoids in rugosa rose hip cuticular wax accounted for approximately 15% of the chloroform-soluble wax extract in very young fruit and increased progressively and steadily during fruit development, reaching almost 42% in ripened rosehips, meaning they increased 2.6-fold from the stage of the very young fruit to the stage of full maturation. Triterpenoid acids were the most prevalent fraction at every stage of fruit development, their content ranging between 72% and 76% of all identified compounds, with the highest levels (76%) detected in young fruits. Ursolic acid was the most prominent constituent, ranging from 68% to 72% of the fraction of acids, with the second most abundant being oleanolic acid (15% of acids in ripened hips). The ratio of ursolic to oleanolic acid increased slightly during rose hip development, where in very young fruit it equaled 3.4:1, and in the fully ripened fruit it reached 4.5:1. Ursane-type acids generally predominated in rugosa rose hip cuticular wax, since the third most predominant acid was corosolic acid (7.7% of acids in ripened hips). It should be noticed that the content of corosolic acid increased sharply (more than 20-fold) during fruit development. Pomolic acid (another ursane-type acid) appeared in fully developed, not yet ripened rose hips and its content also increased markedly (11-fold) during fruit maturation, while oleanane-type maslinic acid was found in very small amounts only in ripened fruit.

The content of neutral triterpenoids constituted from 8% to 13% of the total triterpenoids in cuticular waxes of very young rose hips and ripened fruit, respectively. The content of this fraction increased progressively during fruit development to almost 5-fold higher in the waxes of ripened fruit as compared to the levels detected in very young rose hips. Ursolic aldehyde was the prevailing compound among the neutral triterpenoids, constituting 40–50% of this fraction.

In turn, steroids were the only fraction of compounds identified in cuticular wax of rugosa rose hips that did not increase during fruit development. In very young rose hips, steroids constituted 13% of the total content of identified compounds. During the first stage of fruit growth, the content of steroids decreased markedly to the level of 7.5% of the total triterpenoid content, then increased more than 2-fold in the fully developed but not yet ripened fruit. Finally, the content of steroids constituted 10% of the total triterpenoid content in mature rose hips. Sitosterol was the main phytosterol, constituting approximately 70% of the steroid fraction.

The content of the triterpenoid ester fraction was the lowest in cuticular wax of rugosa rose hips, not exceeding 3% of the total triterpenoid fraction. It increased 3.5-fold during fruit development, particularly sharply in the last stage of fruit growth and maturation. The prevailing compounds were esters of ursolic acid (approximately 60% of the fraction).

Changes in the content of fractions of triterpenoids, steroids and esters in cuticular waxes during rugosa rose fruit development are shown in Figure S1.

### 2.2. Black Chokeberry cv. Galicjanka (Aronia Melanocarpa (Michx.) Elliot)

**Figure 3:**
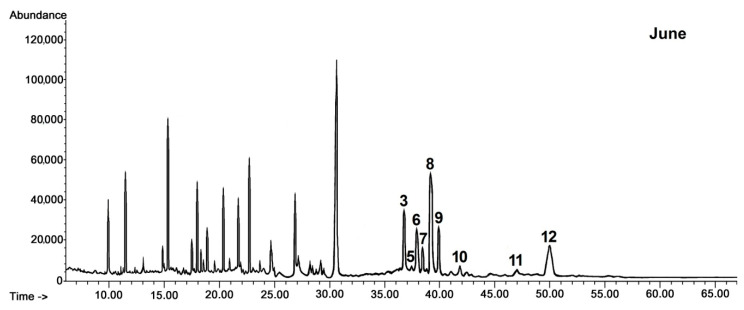

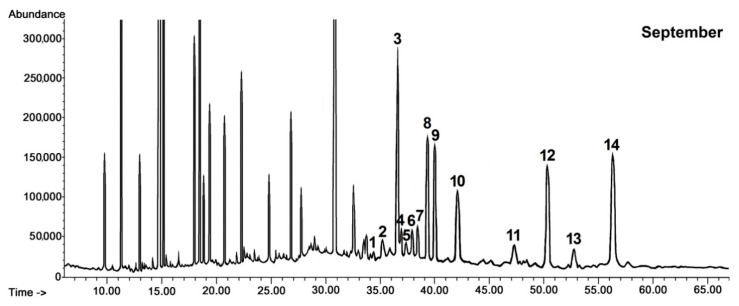
GC-FID chromatograms of the fraction of steroids and neutral triterpenoids obtained from chloroform extracts of cuticular waxes of black chokeberry fruit harvested in two developmental stages (young fruit in June, and ripened fruit in September). 1—campesterol, 2—stigmasterol, 3—sitosterol, 4—sitostanol, 5—cycloartanol, 6—β-amyrin, 7—α-amyrenone, 8—α-amyrin/lupeol, 9—tremulone, 10—sitostanol, 11—oleanolic aldehyde, 12—ursolic aldehyde, 13—erythrodiol, 14—uvaol. Peaks not numbered were associated with aliphatic compounds.

Only a few triterpenoid compounds were found in cuticular wax of black chokeberry fruit harvested in late May and June at the stage of the very young and young growing fruit, whereas the composition of the identified constituents was significantly enriched in fully developed, not yet ripened fruit (harvested in July) and mature chokeberries (harvested in September). The composition of pentacyclic triterpenoids in the cuticular wax of black chokeberry was found to be based exclusively on the compounds with ursane, oleanane and lupane types of carbon skeletons, as was noticed in the cuticular wax of rugosa rose hips. The composition of the fraction of neutral triterpenoids was richer, containing, apart from the previously identified compounds (monohydroxy alcohols α- and β-amyrins, lupeol, ketone α-amyrenone, oleanolic and ursolic aldehydes), also dihydroxy alcohols of oleanane, ursane and lupane types, including erythrodiol (olean-12-ene-2,28-diol), uvaol (urs-12-ene-3,28-diol) and betulin (lup-20(29)-ene-3,28-diol), respectively. Nevertheless, dihydroxy alcohols appeared in black chokeberry cuticular wax only in fully developed fruit and were not found at the earliest stages of fruit development. The characteristic pattern of changes in the content of the neutral triterpenoids in cuticular wax during fruit development of black chokeberry is presented in chromatograms shown in Figure 3.

The identified triterpenoid acids as well as various acid derivatives (3-oxo-analogs, analogs with an additional double bond in position 2 and dihydroxy analogs) belong only to oleanane and ursane groups, whereas betulinic acid (lupane-type) was not detected in fruits of the cultivar “Galicjanka”, though small amounts of betulinic acid were detected in cuticular waxes of another black chokeberry cultivar, “Nero” [21]. In addition to free acids, some esters of relatively high polarity (oleanolic acid methyl ester, as well as acetates of oleanolic and ursolic acids) were identified in black chokeberry cuticular wax. These esters were not found in the typical fractions of low-polar esters (Section 4.3.2), but acetates cofractionated with the fraction of free acids, and the oleanolic acid methyl ester cofractionated with neutral triterpenoids, as described in previous studies [9,15]. Some compounds including 3-oxo-oleanolic acid, oleanolic acid acetate and maslinic and pomolic acids were not present in very young chokeberries but appeared in the later stage of fruit growth.

Besides the pentacyclic compounds, several steroids were also identified in black chokeberry cuticular wax, including basic phytosterols such as campesterol, stigmasterol and sitosterol accompanied by its saturated form sitostanol (syn. stigmastanol, stigmastan-3-ol), saturated sterol precursor cycloartanol (9β,19-cyclo-lanostan-3β-ol) and two ketones: tremulone and sitostenone (stigmast-4-en-3-one). Thus, the composition of the steroid fraction of black chokeberry cuticular wax was more complex than that of rugosa rose hips. However, the entire profile of steroids was identified only in cuticular waxes of ripened fruit, whereas in very young chokeberries, only sitosterol and tremulone were found. The fraction of low-polar esters, analyzed after hydrolysis, contained esters of oleanolic and ursolic acids, α-amyrin/lupeol, β-amyrin and sitosterol.

The results of the quantification of individual compounds are presented in Table 2. The general accumulation pattern of the total content of triterpenoids in the cuticular wax of the black chokeberry can be characterized as consistently increasing, although with various rates during specific stages of fruit development.

The total content of steroids and triterpenoids increased more than 3-fold over the full cycle of fruit maturation, from 14% in chloroform wax extract mass from very young black chokeberry fruit (harvested in May) to 44% in wax extract mass from fully ripened fruit (harvested in September).

Triterpenoid acids comprised the greatest fraction at all developmental stages, constituting 96% of all the triterpenoids during fruit growth and only slightly lower (91%) in ripened fruit. The most predominant triterpenoid acid was ursolic acid, ranging from 67% to 51% of the acid fraction in young and ripened fruit, respectively, followed by oleanolic acid, with an approximate 3:1 ratio of ursolic to oleanolic acid. However, the profile of the fraction of triterpenoid acids changed markedly during black chokeberry fruit development in contrast to other fruits, with very sharply increasing amounts of ursolic and oleanolic acid acetates (71-fold and 31-fold, respectively) as well as dihydroxy analogs of ursolic acid (corosolic and pomolic acids, the latter of which increased 43-fold).

The content of neutral triterpenoids was relatively low in black chokeberry cuticular waxes, reaching only 2.6% of the total triterpenoid content in ripened fruit (approximately 1% of wax extract mass). The most prevailing compounds in this fraction in young fruit were α-amyrin and lupeol (51% of the total fraction); however, during fruit development, growing amounts of ursolic aldehyde (up to 22%) and uvaol (up to 33%) were detected during fruit development. Dihydroxy alcohols of triterpenoids with oleanane, lupane and ursane types of the carbon skeleton were not detected in cuticular waxes of young fruit; however these appeared during the stage of maturation.

The content of steroids was extremely low in cuticular waxes of very young and young growing black chokeberries (0.28% and 0.6% of the total triterpenoid fraction, respectively, contributing approximately 0.04% and 0.16% of the wax extract mass). After the stage of intensive fruit growth, the steroid content increased progressively to 2.5% (a 28-fold increase) of the total triterpenoid content (similarly to the fraction of neutral triterpenoids, i.e., approximately 1% of the wax extract mass). Sitosterol was the prevailing compound (70% of the fraction in very young fruit, 45% in ripened chokeberries).

The content of the fraction of esters was relatively high in black chokeberry fruit cuticular wax, increasing more than 5-fold during fruit development and reaching 3% of the wax extract mass in ripened fruits. The prevailing compounds were esters of ursolic and oleanolic acids, which comprised approximately 55% and 20% of the fraction, respectively.

Changes in the content of fractions of triterpenoids, steroids and esters in cuticular waxes during black chokeberry var. Galicjanka fruit development are shown in Figure S2.

### 2.3. Apple (Malus Domestica Borkh.) var. Antonovka

As observed in rugosa rose and black chokeberry, the profile of triterpenoids in Antonovka apple consisted exclusively of the typical representatives of oleanane-, ursane- and lupane-type groups. Among the neutral triterpenoids, amyrins, lupeol, oleanolic and ursolic aldehydes, as well as dihydroxy alcohols such as erythrodiol, uvaol and botulin, were detected. The fraction of triterpenoid acids had a complex composition similar to that identified in rugosa rose, again featuring the basic representatives of oleanane, ursane and lupane skeletons, including oleanolic, ursolic and betulinic acid, in addition to numerous derivatives of oleanolic and ursolic acids, such as 3-oxo-analogs, analogs with an additional double bond in position 2, 2,3-dihydroxy analogs (such as maslinic and corosolic acids) and 3,19-dihydroxyursolic acid, mainly pomolic acid. The profile of steroids also resembled the steroid composition identified in the cuticular wax of rugosa rose hip, featuring four typical phytosterols (campesterol, isofucosterol, sitosterol and stigmasterol) and the steroid ketone tremulone, as well as one compound not identified previously in rugosa rose: cycloartanol.

The results of the quantification of individual compounds are presented in Table 3. The total content of identified triterpenoids and steroids was relatively high, starting from approximately 25% of chloroform-soluble wax extract in very young fruits and increasing 2-fold during the stage of intensive fruit growth, reaching almost 50% in developed but not fully ripened apples, followed by a slight (5%) decrease during the final step of maturation. Triterpenoid acids were the prevailing fraction, constituting 98% of all identified compounds in young fruit, 96% in developed fruit and 94% in ripe apples. Ursolic acid was the most prominent constituent, making up approximately 66% of acids, followed in second place by oleanolic acid, making up 22% of acids, and, thirdly, corosolic acid, making up 10% of acids. As in rose hip and black chokeberry fruit cuticular waxes, ursane-type acids predominated; however, the ratio of ursolic to oleanolic acid decreased during apple fruit development, from 5:1 in very young fruit to only 3:1 in ripened Antonovka apples. The representative of lupane-type compounds, betulinic acid, was not detected in cuticular waxes of very young fruit but appeared during the stage of intensive fruit growth. Its level increased almost 2-fold, then slightly decreased (by 7%) during fruit maturation, reflecting the general trend observed for the content of triterpenoid acids. Simultaneously, the content of oleanane-type maslinic acid decreased 2.5-fold during apple fruit growth, the only acid to display such a consistent tendency to decrease.

Fractions of the neutral triterpenoids and steroids were in very low abundance in Antonovka apple cuticular wax, with neither exceeding 0.8% of the wax extract mass. The steroid and neutral triterpenoids increased approximately 3-fold from the stage of very young fruits harvested in May soon after fruit formation to the stage of almost developed fruits harvested in late July. Lupeol/α-amyrin and ursolic aldehyde were the most abundant neutral triterpenoids, constituting 50% and 27% of this fraction in ripened apples, respectively. The content of a ursane-type dihydroxy alcohol, uvaol, increased 4-fold during all stages of fruit development. The other dihydroxy alcohols, oleanane-type erythrodiol and lupane-type betulin, were not present in the very young fruits: erythrodiol appeared during the stage of fruit growth and betulin only at the last stage of fruit maturation. Sitosterol was the most prevailing steroid, comprising 77% of this fraction, and its content increased 3-fold over the full period of fruit development and maturation. However, the content of campesterol, stigmasterol and cycloartanol decreased during the stage of intensive fruit growth from late May to late July by 30% and 40%, in the case of campesteol and stigmasterol, respectively. The content of esters in Antonovka apple cuticular wax was very low, constituting approximately 0.3% of the wax extract mass in ripened fruits. The prevailing compounds were esters of ursolic acid, making up 60% of this fraction.

Changes in the content of fractions of triterpenoids, steroids and esters in cuticular waxes during apple var. Antonovka fruit development are shown in Figure S3.

## 3. Discussion

The results obtained in the present study, as well as data available in several previous reports [8,9,22], reveal noticeable differences in the triterpenoid composition of fruit cuticular wax across species and cultivars. Numerous strategies of triterpenoids synthesis and their deposition in cuticular wax exist in various fruit species, from their significant abundance in the wax composition, as demonstrated in bilberry, lingonberry and strawberry tree fruits of Ericaceae [9], grapes of Vitaceae [15] and currently investigated black chokeberry, apple and rugosa rose of Rosaceae, to trace amounts present in honeysuckle of Caprifoliaceae [9]. Moreover, even among fruits exhibiting an abundance of triterpenoids, much variation was observed in the profiles of these compounds with respect to the prevalence of acids (such as in bilberry and lingonberry of Ericaceae [9], as well as fruits of Rosaceae described in this study) in relation to alcohols (such as in strawberry tree fruit of Ericaceae [9] or the very well-known examples of Solanaceae such as tomato [22]). Since apple is a commercially important crop, the composition of triterpenoid acids in apple cuticular wax is one of the best characterized among all fruits. Numerous apple varieties grown in many regions of the world were investigated in terms of their cuticular wax composition [23,24,25,26,27]. Ursolic and oleanolic acids have been detected in all apple varieties analyzed so far; however, they are not always the prevailing compounds. In some varieties, betulinic acid predominates, while in others, maslinic acid or even relatively rare acids such as annurcoic acid and pomaceic acid [28] are the predominant acids. A recent report concerning the content of triterpenoid acids in apples revealed that pomolic, euscaphic, maslinic and ursolic acids were the most abundant triterpenoids in peels of ancient apple varieties cultivated in the Friuli Venezia Giulia region in Northeast Italy, while ursolic and oleanolic acids were more prevalent in commercial fruits, such as Golden Delicious, Red Delicious, Granny Smith and Royal Gala [28]. However, although some similarities in composition among phylogenetically close species seem to exist, e.g., the prevalence of ursolic acid over oleanolic acid (observed in fruits investigated in the present study, as well as in other Rosaceae fruits such as quince, loquat, pear or peach [29]) and the characteristic ratio of triterpenoid acids vs. triterpenoid alcohols distinguishing the Vaccinium and Arbutus genera [9], no clear correspondence of the triterpenoid composition pattern with fruit types (such as berries, pome or other pseudo-fruits) could be shown on the basis of existing knowledge.

Our understanding of the relevance of triterpenoids for fruit quality traits is still preliminary. Some studies have shown that softening rates are highly relevant to the content of ursolic acid [30], while the occurrence of micro-cracking correlates with the high content of betulinic acid [26]. Suberized apple peel, found in partially and fully russeted old heritage varieties, was found to contain higher concentrations of lupane derivatives, including betulinic acid, than waxy skinned cultivars [25,26]. Triterpenoids represent a more polar class of wax constituents, and their occurrence is often considered as a feature increasing the wetting characteristics and water permeability of the fruit surface [16]. Thus, triterpenoids can play a major role in regulating fruit cuticle permeability and susceptibility to pathogens. Further studies are still required to understand how the deposition pattern of triterpenoids in cuticular wax can influence the fruit quality and storability characteristics.

During the development of fruits selected for this study, all belonging to the Rosaceae family, the progressive increase in the content of triterpenoids, as well as the enrichment of the composition of these compounds in the cuticular wax, could be observed. The main profile of identified compounds was rather similar across all three species, with triterpenoids of ursane, oleanane and lupane types of the carbon skeleton, high levels of ursolic acid and the typical composition of steroids. However, some specific differences in the patterns of triterpenoid and steroid accumulation during fruit development and maturation between studied fruits were noticed.

In rugosa rose hip cuticular wax, a constant increase in the total content of triterpenoids was observed, including acids and neutral triterpenoids (the latter accumulated at the highest level among fruits investigated in this study). The changes in the profile of triterpenoids comprised the gradual appearance of dihydroxy acids. Rugosa rose was the only fruit in which the characteristic accumulation pattern of steroids described previously for other fruits from the Ericaceae and Caprofoliaceae families [9] (meaning a decrease during young fruit intensive growth and an increase during maturation) was noticed.

In turn, the most characteristic pattern of changes in triterpenoid content during black chokeberry fruit development was the increasing esterification (i.e., acetylation) of the predominating ursolic and oleanolic acids, as well as a gradual appearance of dihydroxy alcohols and dihydroxy acids. The total level of triterpenoid acids in black chokeberry decreased slightly during fruit maturation, while the content of steroids started out very low in young fruits and increased markedly during fruit maturation.

The accumulation of steroids and triterpenoids in cuticular waxes of Antonovka apple increased steadily during fruit development, except during the last step of growing and maturation, when the content of triterpenoid acids started to decrease slightly. The content of dihydroxy alcohols, not present in very young fruit, increased during the last stage of maturation. The levels of total sterols increased during all periods of development and maturation, mainly due to constantly increasing levels of sitosterol.

The Rosaceae family is one of the major angiosperm families, with many plant species bearing various fruits as follicles, capsules, nuts, achenes, drupes and accessory fruits, such as the pome of an apple or the hip of a rose. For this study, three plant species bearing edible accessory fruits (pseudo-fruits) were chosen. The reported studies on Rosaceae species bearing other types of fruit, such as a previous study on sweet cherry [16] or the current study on nectarine (*Prunus persica* L. Batsch) [31], described the various patterns of triterpenoid deposition during fruit development and ripening. In nectarine fruit, an intense accumulation of triterpenoids during initial fruit growth, followed by their decrease at the end of endocarp lignification, and a final increase in the level of hydroxylated triterpenoids until maturity were noticed [31].

The variability observed in cuticular wax composition, cuticle architecture and cuticular properties clearly indicates that the cuticle and cuticular waxes are features of crop plants that can be subjected to plant breeding in order to improve some agronomically important traits. However, according to Lara et al. [7], “devising a generalized model of cuticle structure, together with a comprehensive atlas of differences between organisms, to translate this knowledge into a practical understanding of the resulting biophysical properties” is still rather far to be achieved. Nevertheless, studies on the function of the fruit cuticle, the composition of cuticular waxes and their dynamics during fruit development and ripening are becoming increasingly required for contemporary agriculture. Development of crops which are less susceptible to water loss and pathogen infection can be crucial to face the consequences of limited water supply imposed by climate change, as well as a growing demand to improve agricultural production to avoid post-harvest spoilage [5].

Another important implication of research into the triterpenoid content in fruit cuticular waxes is the potential for recycling of industrial fruit residues as a valuable source of bioactive compounds for functional food, pharmaceuticals and cosmetics [22,32]. Industrial leftover material, such as peels from juice production, provides raw material for isolating fruit wax compounds, such as value-added wax that might be used for packaging and nanocoating [4]. Thus, paradoxically, fruit cuticles can potentially offer a natural alternative for synthetic waxes.

## 4. Materials and Methods

### 4.1. Plant Material

Fruits of the black chokeberry *Aronia melanocarpa* (Michx.) Elliot (*Rosaceae*) variety “Galicjanka”, apple *Malus domestica* Borkh. (*Rosaceae*) variety “Antonovka” and rugosa rose *Rosa rugosa* Thunb. (*Rosaceae*) were collected from a private orchard in Stare Bosewo, central Poland (52°460 N, 21°332 E). The collection of fruits from plants growing in the same habitat (thus the same type of soil, climate conditions, insolation and water supply) helped to avoid the possible divergences resulting from different environmental conditions. Saplings of *A. melanocarpa, R. rugosa* and *M. domestica* were purchased from the Polish vegetable seed farming and nursery enterprise “PNOS” and cultivated in an open field.

All fruits were collected at four different stages: very young fruit (approximately one week after flowering), young fruit, fully developed but unripe fruit and mature fruit. To avoid the impact of plant inter-individual variability, fruit samples were harvested at every developmental stage from the same plants. Replicates of approximately 2 g samples of black chokeberry fruit were prepared from pooled sample sets of 10–15 g. The replicates of 5–10 intact and healthy fruits of *M. domestica* and *R. rugosa* were selected from harvested pooled sample sets of approximately 20 fruits per set.

### 4.2. Chemicals and Standards

All solvents: chloroform, diethyl ether, methanol, and chemicals: CH_3_COOH, KOH, NaOH (purchased from Chempur, Piekary Śląskie, Poland), used for extraction and analysis were of analytical grade. Authentic standards of α-amyrin and ursolic acid methyl ester were purchased from Roth (Karlsruhe, Germany); β-amyrin, lupeol, uvaol, oleanolic acid, campesterol, sitosterol and stigmasterol were purchased from Sigma-Aldrich (Steinheim, Germany).

### 4.3. Extraction and Fractionation

#### 4.3.1. Extraction of Waxes

The procedure of wax extractions was reported previously [9,15,19,20]. Each sample of entire fruits was extracted by incubation in an appropriate volume of chloroform (approximately 10 times more than the volume of extracted plant material) with gentle stirring for 30 s at room temperature. All experiments were performed in three replicates. The extracts were decanted, filtered and evaporated to dryness under a gentle stream of nitrogen; methods and protocols will be described in detail, while well-established methods will be briefly described and appropriately cited.

#### 4.3.2. Fractionation of Chloroform Extracts

Evaporated chloroform extracts were fractionated by adsorption preparative TLC on 20 × 20 cm glass plates coated manually with silica gel 60H (Merck, Darmstad, Germany). The solvent system chloroform/methanol 97:3 (*v/v*) was applied for developing. Three fractions were obtained as described earlier: free (non-esterified) steroids and triterpenoids, triterpenoid acids and steroid/triterpenoid esters [15,19]. Fractions were eluted from the gel in diethyl ether. Subsequently, fractions containing free neutral triterpenes and sterols (*R_f_* 0.3–0.9) were directly analyzed by GC-MS, fractions containing triterpene acids (*R_f_* 0.2–0.3) were methylated with diazomethane and fractions containing triterpenoid (triterpene and sterol) esters (*R_f_* 0.9–1) were subjected to alkaline hydrolysis. The average recovery of α-amyrin, uvaol, stigmasterol and ursolic acid methyl ester from preparative TLC plates was 98.6%, 97.2%, 98.9% and 96.1%, respectively [15].

### 4.4. Derivatization of Triterpenoid Acids

Nitrosomethylurea (2.06 g) was added to a mixture of 20 mL of diethyl ether and 6 mL of 50% aqueous KOH, and the organic layer was then separated from the aqueous layer. Samples containing triterpenoid acids were dissolved in 2 mL of the obtained solution of diazomethane in diethyl ether and held at 2 °C for 24 h.

### 4.5. Alkaline Hydrolysis

The ester fraction was subjected to alkaline hydrolysis with 10% NaOH in 80% MeOH at 80 °C for 3 h. Subsequently, 5 volumes of water were added to each hydrolysate, the pH was neutralized with 5% CH_3_COOH and the obtained mixtures were extracted with diethyl ether (3 × 10 mL). These extracts were fractionated by preparative TLC as described above, then fractions containing free triterpene alcohols and sterols were directly analyzed by GC-MS, while triterpene acid fractions were methylated prior to this analysis.

### 4.6. Identification and Quantification of Steroids and Triterpenoids by GC-MS/FID

An Agilent Technologies 7890A gas chromatograph (Perlan Technologies, Warszawa, Poland) equipped with a 5975C mass spectrum detector was used for qualitative and quantitative analyses. Samples dissolved in diethyl ether/methanol (5:1, *v/v*) were applied (in a volume of 1–4 µL) using 1:10 split injection. The column used was a 30 m × 0.25 mm i.d., 0.25 μm, HP-5MS UI (Agilent Technologies, Santa Clara, CA). Helium was used as the carrier gas at a flow rate of 1 mL/min. The separation was made with the following temperature program: initial temperature of 160 °C held for 2 min, then increased to 280 °C at 5 °C/min, and the final temperature of 280 °C held for a further 44 min. The other employed parameters were as follows: inlet and FID (flame ionization detector, part of 7890A chromatograph) temperature 290 °C; MS transfer line temperature 275 °C; quadrupole temperature 150 °C; ion source temperature 230 °C; EI 70 eV; *m/z* range 33–500; FID gas (H_2_) flow 30 mL/min (hydrogen generator HydroGen PH300, Peak Scientific, Inchinnan, UK); and air flow 400 mL/min. Individual compounds were identified by comparing their mass spectra with spectral libraries (Wiley 9th ED. and NIST 2008 Lib. SW Version 2010; Agilent Technologies, Santa Clara, CA), previously reported data and the results of earlier experiments, as well as by comparison of their retention times and corresponding mass spectra with those of authentic standards, where available. Retention times and characteristic ions of the mass spectra of the compounds identified in the present study are presented in Table S1. Quantitation was conducted with an FID detector and performed using an external standard method based on calibration curves determined for authentic standards of ursolic acid methyl ester, α-amyrin and stigmasterol [15,19].

### 4.7. Statistical Analysis of Data

All experiments were performed in triplicate. Data are presented as the means ± standard deviation of three independent samples analyzed in triplicate. The data were subjected to one-way analysis of variance (ANOVA), and the differences between means were evaluated using Duncan’s multiple-range test. Statistical significance was considered to be obtained at *p* < 0.05.

## 5. Conclusions

The results obtained in this study reveal that the observed changes in triterpenoid content during fruit growth and maturation are not as simple as a decrease or increase in accumulation of individual compounds, but rather reflect deeper modifications of metabolic pathways, particularly involving the stimulation of such processes as hydroxylation and esterification. The increase in levels of hydroxylated and/or esterified triterpenoid derivatives can influence the properties of cuticular waxes by changing the interactions of these compounds with complementary functional groups of other constituents (such as free fatty acids, aliphatic long-chain primary and secondary alcohols and aldehydes), thus leading to modification of a degree of molecular order and spatial arrangement of the wax matrix. Although this phenomenon cannot be treated as universal for all fruits which display a tendency toward increased accumulation of triterpenoids during fruit development, it deserves attention since it can be responsible for some important changes in fruit surface quality occurring during fruit maturation, such as impaired barrier properties or formation of microcracks, leading to fruit shriveling and susceptibility to pathogens and mold.

## Figures and Tables

**Table 1:** Content of triterpenoids in cuticular waxes during rugosa rose (*Rosa rugosa*) fruit development (very young fruit harvested in June, young fruit in July, fully developed but not ripened fruit in August and ripened fruit in September).

Compound	Content [mg/g Wax Extract ± SD (Standard Deviation)]			
	June	July	August	September
α-amyrin/lupeol	2.97 ± 0.31 ^a^	5.21 ± 0.55 ^b^	9.04 ± 1.02 ^c^	12.16 ± 1.40 ^d^
β-amyrin	1.17 ± 0.12 ^a^	3.90 ± 0.42 ^b^	5.58 ± 0.64 ^c^	7.92 ± 0.84 ^d^
α-amyrenone	1.02 ± 0.10 ^a^	3.48 ± 0.36 ^b^	5.17 ± 0.53 ^c^	6.08 ± 0.62 ^d^
oleanolic aldehyde	0.79 ± 0.08 ^a^	4.06 ± 0.48 ^b^	5.95 ± 0.62 ^c^	6.50 ± 0.70 ^c^
ursolic aldehyde	5.95 ± 0.62 ^a^	13.63 ± 1.50 ^b^	17.54 ± 2.02 ^c^	21.88 ± 2.42 ^d^
Sum of neutral triterpenoids	11.90	30.28	43.28	54.54
olean-2,12-dien-28-oic acid	0.86 ± 0.09 ^a^	0.92 ± 0.10 ^a^	1.06 ± 0.12 ^a^	1.15 ± 0.12 ^a^
ursa-2,12-dien-28-oic acid	3.12 ± 0.32 ^a^	4.36 ± 0.48 ^b^	5.59 ± 0.65 ^c^	6.43 ± 0.67 ^c^
3-oxo-oleanolic acid	0.64 ± 0.08 ^a^	0.14 ± 0.02 ^b^	0.10 ± 0.01 ^b^	0.12 ± 0.01 ^b^
oleanolic acid	24.16 ± 2.52 ^a^	27.67 ± 3.01 ^a^	41.92 ± 4.68 ^b^	46.94 ± 5.22 ^b^
3-oxo-ursolic acid	1.12 ± 0.14^a^	1.25 ± 0.15 ^a^	1.32 ± 0.14 ^a^	1.97 ± 0.20 ^b^
ursolic acid	82.02 ± 10.06 ^a^	110.09 ± 14.50 ^b^	157.68 ± 18.24 ^c^	209.80 ± 22.06 ^d^
betulinic acid	1.59 ± 0.16 ^a^	3.83 ± 0.41 ^a^	5.12 ± 0.62 ^c^	8.11 ± 0.90 ^d^
maslinic acid	n.d.	n.d.	n.d.	0.96 ± 0.11 ^a^
corosolic acid	1.15 ± 0.10^a^	4.16 ± 0.44 ^b^	5.98 ± 0.64 ^c^	23.55 ± 2.50 ^d^
pomolic acid	n.d.	n.d.	0.55 ± 0.07 ^a^	6.12 ± 0.62 ^b^
Sum of triterpenoid acids	114.66	152.42	219.32	305.15
campesterol	0.41 ± 0.05 ^a^	0.15 ± 0.01 ^b^	0.54 ± 0.06 ^c^	0.69 ± 0.08 ^d^
cholesterol	0.09 ± 0.01 ^a^	0.04 ± 0.01 ^b^	0.13 ± 0.01 ^c^	0.28 ± 0.03 ^d^
isofucosterol	2.44 ± 0.26 ^a^	1.98 ± 0.22 ^a^	5.47 ± 0.61 ^c^	6.12 ± 0.64 ^c^
sitosterol	14.52 ± 1.62 ^a^	10.95 ± 1.10 ^b^	23.31 ± 2.35 ^c^	32.75 ± 3.50 ^d^
stigmasterol	0.11 ± 0.01 ^a^	0.06 ± 0.01 ^b^	0.19 ± 0.02 ^c^	0.22 ± 0.02 ^c^
tremulone	2.90 ± 0.32 ^a^	1.95 ± 0.23 ^b^	3.72 ± 0.38 ^c^	5.25 ± 0.54 ^d^
Sum of steroids	20.47	15.13	33,36	45.31
esters:				
α-amyrin/lupeol	0.14 ± 0.02 ^a^	0.55 ± 0.06 ^b^	0.64 ± 0.08 ^b^	0.98 ± 0.10 ^c^
oleanolic acid	0.29 ± 0.03 ^a^	0.62 ± 0.07 ^b^	0.93 ± 0.10 ^c^	1.36 ± 0.14 ^d^
ursolic acid	2.12 ± 0.24 ^a^	3.08 ± 0.34 ^b^	4.51 ± 0.47 ^c^	7.24 ± 0.78 ^d^
sitosterol	0.92 ± 0.10 ^a^	0.12 ± 0.01 ^b^	1.57 ± 0.15 ^c^	2.45 ± 0.27 ^d^
Sum of esters	3.47	4.37	7.65	12.03
Total	150.5	202.2	303.61	417.03

Results are referenced to wax extract mass and expressed as the mean ± SD of three independent samples analyzed in triplicate. Results in rows not sharing a common letter are significantly different (*p* < 0.05).

**Table 2:** Content of triterpenoids in cuticular waxes during black chokeberry (*Aronia melanocarpa*) var. Galicjanka fruit development (very young fruit harvested in May, young fruit in June, fully developed but not ripened fruit in late July and ripened fruit in September).

Compound	Content [mg/g Wax Extract ± SD (Standard Deviation)]			
	May	June	July	September
α-amyrin/lupeol	1.18 ± 0.22 ^a^	1.96 ± 0.24 ^b^	2.39 ± 0.26 ^b^	2.55 ± 0.37 ^b^
β-amyrin	0.35 ± 0.04 ^a^	0.42 ± 0.05 ^a^	0.64 ± 0.08 ^b^	0.79 ± 0.08 ^b^
α-amyrenone	0.09 ± 0.01 ^a^	0.27 ± 0.02 ^b^	0.59 ± 0.07 ^c^	0.84 ± 0.92 ^d^
erythrodiol	n.d.	n.d.	0.14 ± 0.0 ^a^	0.35 ± 0.03 ^b^
betulin	n.d.	n.d.	0.03 ± 0.01 ^a^	0.04 ± 0.01 ^a^
oleanolic aldehyde	0.11 ± 0.01 ^a^	0.25 ± 0.03 ^b^	0.33 ± 0.04 ^c^	0.45 ± 0.05 ^d^
ursolic aldehyde	0.58± 0.06 ^a^	0.96 ± 0.10 ^b^	1.69 ± 0.18 ^c^	3.81 ± 0.40 ^d^
uvaol	n.d.	n.d.	2.14 ± 0.24 ^a^	2.56 ± 0.28 ^a^
Sum of neutral triterpenoids	2.31	3.86	7.95	11.39
olean-2,12-dien-28-oic acid	2.05 ± 0.23 ^a^	4.46 ± 0.48 ^b^	1.01 ± 0.09 ^c^	1.31 ± 0.12 ^c^
ursa-2,12-dien-28-oic acid	8.16 ± 0.84 ^a^	15.65 ± 1.70 ^b^	2.17 ± 0.25 ^c^	2.23 ± 0.30 ^c^
3-oxo-oleanolic acid	n.d.	0.46 ± 0.06 ^a^	1.33 ± 0.15 ^b^	1.38 ± 0.16 ^b^
oleanolic acid	31.54 ± 3.22 ^a^	54.42 ± 5.60 ^b^	71.08 ± 7.15 ^c^	65.03 ± 6.13 ^c^
oleanolic acid methyl ester	0.25 ± 0.03 ^a^	0.42 ± 0.05 ^b^	1.08 ± 0.12 ^c^	1.29 ± 0.14 ^c^
oleanolic acid acetate	n.d.	0.69 ± 0.07 ^a^	20.60 ± 2.12 ^b^	21.89 ± 2.30 ^b^
3-oxo-ursolic acid	0.14 ± 0.01 ^a^	4.42 ± 0.48 ^b^	5.51 ± 0.60 ^b^	5.59 ± 0.62 ^b^
ursolic acid	92.68 ± 10.44 ^a^	164.63 ± 20.05 ^b^	227.48 ± 25.06 ^c^	207.27 ± 22.16 ^c^
ursolic acid acetate	0.96 ± 0.10 ^a^	5.75 ± 0.60 ^b^	59.22 ± 7.14 ^c^	68.55 ± 8.01 ^c^
maslinic acid	n.d.	0.38 ± 0.01 ^a^	1.12 ± 0.14 ^b^	1.15 ± 0.16 ^b^
corosolic acid	0.69 ± 0.08 ^a^	3.55 ± 0.40 ^b^	7.19 ± 0.85 ^c^	7.37 ± 0.90 ^c^
pomolic acid	n.d.	0.48 ± 0.06 ^a^	14.69 ± 1.60 ^b^	20.95 ± 2.35 ^c^
Sum of triterpenoid acids	136.47	255.31	412.48	404.01
campesterol	n.d	n.d.	n.d.	0.12± 0.02
cycloartanol	n.d.	0.21 ± 0.02 ^a^	0.38 ± 0.04 ^b^	0.45 ± 0.05 ^b^
sitosterol	0.28 ± 0.03 ^a^	0.87 ± 0.10 ^b^	3.55 ± 0.38 ^c^	5.05± 0.54 ^d^
sitostanol	n.d.	n.d.	0.43 ± 0.05 ^a^	0.84 ± 0.08 ^b^
sitostenone	n.d.	0.17 ± 0.01 ^a^	0.66 ± 0.08 ^b^	1.69± 0.11 ^c^
stigmasterol	n.d.	n.d.	0.14 ± 0.01 ^a^	0.68± 0.07 ^b^
tremulone	0.12 ± 0.01 ^a^	0.41 ± 0.04 ^b^	1.70 ± 0.19 ^c^	2.39± 0.25 ^d^
Sum of steroids	0.4	1.66	6.86	11.22
esters:				
α-amyrin/lupeol	0.02 ± 0.01 ^a^	0.08± 0.01 ^b^	0.22± 0.01 ^c^	1.87± 0.21 ^d^
β-amyrin	n.d.	n.d.	0.09± 0.01 ^a^	0.88± 0.11 ^b^
oleanolic acid	0.92± 0.10 ^a^	1.70 ± 0.20 ^b^	1.85 ± 0.20 ^b^	2.92 ± 0.30 ^c^
ursolic acid	1.75± 0.18 ^a^	2.54 ± 0.26 ^b^	3.96 ± 0.42 ^c^	7.98 ± 0.82 ^d^
sitosterol	n.d.	n.d.	0.09± 0.01 ^a^	0.84 ± 0.10 ^b^
Sum of esters	2.69	4.32	6.21	14.49
Total	141.87	265.15	433.5	441.11

Results are referenced to wax extract mass and expressed as the mean ± SD of three independent samples analyzed in triplicate. Results in rows not sharing a common letter are significantly different (*p* < 0.05).

**Table 3:** Content of triterpenoids in cuticular waxes during apple (*Malus domestica*) var. Antonovka fruit development (very young fruit harvested in late May, young fruit in June, fully developed but not ripened fruit in July and ripened fruit in September).

Compound	Content [mg/g Wax Extract ± SD (Standard Deviation)]			
	May	June	July	September
α-amyrin/lupeol	0.71 ± 0.04 ^a^	1.03 ± 0.09 ^b^	2.25 ± 0.20 ^c^	2.39 ± 0.25 ^c^
β-amyrin	0.38 ± 0.02 ^a^	0.52 ± 0.02 ^b^	0.62 ± 0.02 ^c^	0.91 ± 0.03 ^d^
erythrodiol	n.d.	0.09 ± 0.01 ^a^	0.14 ± 0.01 ^b^	0.22 ± 0.01 ^c^
betulin	n.d.	n.d.	n.d.	0.08 ± 0.01 ^a^
oleanolic aldehyde	0.06 ± 0.02 ^a^	0.15 ± 0.01 ^b^	0.22 ± 0.01 ^c^	0.25 ± 0.01 ^d^
ursolic aldehyde	1.09 ± 0.05 ^a^	1.54 ±0.11 ^b^	1.97 ± 0.18 ^c^	2.14 ± 0.19 ^c^
uvaol	0.23 ± 0.01 ^a^	0.31 ± 0.01 ^b^	0.57 ± 0.02 ^c^	1.83 ± 0.12 ^d^
Sum of neutral triterpenoids	2.47	3.64	5.77	7.82
olean-2,12-dien-28-oic acid	1.12 ± 0.09 ^a^	1.24 ± 0.12 ^a^	1.48 ± 0.12 ^a^	1.71 ± 0.18 ^a^
ursa-2,12-dien-28-oic acid	4.26 ± 0.42 ^a^	4.00 ± 0.55 ^a^	4.76 ± 0.52 ^a^	5.62 ± 0.60 ^b^
3-oxo-oleanolic acid	0.34 ± 0.01 ^a^	0.42 ± 0.02 ^b^	1.34 ± 0.2 ^c^	2.80 ± 0.32 ^d^
oleanolic acid	28.77 ± 2.15 ^a^	57.59 ± 6.25 ^b^	106.96 ± 14.02 ^c^	96.57 ± 11.13 ^c^
3-oxo-ursolic acid	2.1± 0.15 ^a^	2.43 ± 0.19 ^a^	2.05 ± 0.19 ^a^	5.59 ± 0.62 ^b^
betulinic acid	n.d.	5.44 ± 0.62 ^a^	9.28 ± 1.10 ^b^	8.64 ± 1.02 ^b^
ursolic acid	152.56 ± 22.48 ^a^	239.82 ± 20.06 ^b^	303.45 ± 28.37 ^c^	295.32 ± 30.16 ^c^
maslinic acid	3.32 ± 0.29 ^a^	2.16 ± 0.18 ^b^	1.77 ± 0.12 ^c^	1.32 ± 0.14 ^d^
corosolic acid	42.85 ± 3.90 ^a^	58.39 ± 6.11 ^b^	50.50 ± 4.81 ^b^	37.73 ± 4.05 ^a^
pomolic acid	0.11 ± 0.10 ^a^	0.21 ± 0.09 ^b^	0.32 ± 0.05 ^c^	0.29 ± 0.04 ^d^
Sum of triterpenoid acids	238.43	368.26	480.64	446.95
campesterol	0.18 ± 0.01 ^a^	0.38 ± 0.01 ^b^	0.27 ± 0.01 ^c^	0.40 ± 0.02 ^b^
cycloartanol	0.15 ± 0.01 ^a^	0.45 ± 0.01 ^b^	0.42 ± 0.02 ^a^	0.45 ± 0.03 ^c^
isofucosterol	0.20 ± 0.01 ^a^	0.32 ± 0.02 ^b^	0.39 ± 0.01 ^c^	0.54 ± 0.03 ^b^
sitosterol	1.96 ± 0.20 ^a^	3.29 ± 0.65 ^b^	5.47 ± 0.55 ^c^	5.84 ± 0.52 ^c^
stigmasterol	n.d.	0.15 ± 0.01 ^a^	0.09 ± 0.01 ^b^	0.10 ± 0.01 ^b^
tremulone	0.10 ± 0.01 ^a^	0.08 ± 0.03 ^b^	0.16 ± 0.01 ^b^	0.21 ± 0.01 ^c^
Sum of steroids	2.59	4.67	6.8	7.54
esters:				
α-amyrin/lupeol	0.04 ± 0.01 ^a^	0.10 ± 0.01 ^b^	0.17 ± 0.01 ^c^	0.22 ± 0.01 ^d^
β-amyrin	n.d.	0.02 ± 0.01 ^a^	0.06 ± 0.01 ^b^	0.09 ± 0.03 ^c^
oleanolic acid	0.53 ± 0.01 ^a^	0.70 ± 0.03 ^b^	0.85 ± 0.02 ^c^	0.92 ± 0.02 ^d^
ursolic acid	0.86 ± 0.02 ^a^	1.54 ± 0.12 ^b^	1.96 ± 0.18 ^c^	1.98 ± 0.21 ^c^
sitosterol	n.d.	n.d.	0.01 ± 0.01 ^a^	0.04 ± 0.01 ^b^
Sum of esters	1.43	2.36	3.05	3.25
Total	241.92	382.37	497.53	474.20

Results are referenced to wax extract mass and expressed as the mean ± SD of three independent samples analyzed in triplicate. Results in rows not sharing a common letter are significantly different (*p* < 0.05).

## Supplementary Materials

Supplementary materials can be found at https://www.mdpi.com/1422-0067/21/24/9762/s1. Table S1. Relative retention times and characteristic ions of mass spectra of identified steroids and triterpenoids. Figure S1. Changes in the content of triterpenoids in cuticular waxes during rugosa rose *Rosa rugosa* fruit development. Figure S2. Changes in the content of triterpenoids in cuticular waxes during black chokeberry *Aronia melanocarpa* var. Galicjanka fruit development. Figure S3. Changes in the content of triterpenoids in cuticular waxes during apple *Malus domestica* var. Antonovka fruit development.

## Abbreviations

GC-MS	Gas chromatography–mass spectroscopy
FID	Flame ionization detector
TLC	Thin-layer chromatography
