# Supplementary material for: Variations in Triterpenoid Deposition in Cuticular Waxes during Development and Maturation of Selected Fruits of Rosaceae Family
Source: Int J Mol Sci. 2020 Dec 21;21(24):9762. doi: 10.3390/ijms21249762 (PMC7767361; doi:10.3390/ijms21249762)
Supplement: Supplementary file 1 [file ijms-21-09762-s001.pdf]

Table S1. Retention times and characteristic ions of mass spectra of identified steroids and triterpenoids

Range of Retention Time	Compound	Mass Spectrum *m/z* (Relative Intensity)
31.8–32.0	Cholesterol	386 (26), 107 (50), 105 (48), 91 (57), 81 (54), 79 (46), 69 (47), 57 (87), 55 (73), 43 (100), 41 (55)
34.4–34.5	Campesterol	400 (30), 107 (51), 105 (55), 95 (49), 83 (45), 81 (64), 71 (62), 57 (77), 55 (77), 43 (100), 41 (52)
35.5–35.6	Stigmasterol	412 (36), 145 (64), 107 (52), 95 (100), 83 (66), 81 (90), 78 (60), 69 (67), 67 (85), 55 (69)
37.4–37.6	Sitosterol	414 (29), 145 (54), 107 (59), 105 (60), 95 (54), 91 (49), 81 (57), 57 (68), 55 (70), 43 (100), 41 (44)
37.6–37.8	Sitostanol	416 (31), 215 (82), 109 (58), 107 (83), 95 (81), 93 (64), 81 (84), 69 (60), 57 (64), 55 (81), 43 (100)
38.1–38.2	Cycloartanol	428 (4), 205 (60), 109 (98), 95 (100), 93 (64), 81 (69), 69 (78), 57 (73), 55 (82), 43 (89), 41 (67)
38.6–38.8	β-Amyrin	426 (27), 219 (18), 218 (100), 203 (49), 189 (17), 135 (11), 109 (13), 105 (12), 95 (15), 81 (18), 69 (14)
40.0–40.2	α-Amyrenone	424 (12), 219 (19), 218 (100), 203 (24), 189 (16), 135 (19), 133 (18), 122 (18), 119 (17), 95 (16), 55 (18)
40.4–40.8	α-Amyrin/	426 (4), 218 (100), 203 (20), 189 (36), 135 (35), 121 (32), 109 (32), 107 34), 95 (40), 81 (33), 55 (31)
	Lupeol	426 (18), 207 (67), 189 (90), 135 (83), 121 (80) 109 (85), 121 (80), 95 (100), 93 (87), 81 (86),
40.9–41.1	Tremulone (stigmasta-3,5-dien-7-one)	410 (32), 187 (27), 174 (100), 161 (37), 159 (26), 91 (28), 57 (28), 55 (37), 43 (44), 41 (28)
42.0–42.2	Sitostenone	412 (37), 229 (34), 218 (31), 124 (100), 109 (31), 95 (41), 81 (27), 69 (32), 55 (37), 43 (44)
48.3–48.5	Oleanolic aldehyde	440 (2), 232 (28), 207 (20), 204 (39), 203 (100), 189 (29), 105 (18), 81 (19), 69 (20), 55 (29)
51.4–51.6	Ursolic aldehyde	440 (1), 207 (26), 204 (23), 203 (100), 133 (42), 119 (18), 105 (18), 95 (18), 81 (18), 55 (18), 43 (20)
53.2–53.4	Erythrodiol	442 (1), 204 (17), 203 (100), 133 (7), 119 (9), 105 (8), 95 (9), 93 (8), 81 (8), 69 (9), 55 (8)
56.3–56.5	Uvaol	442 (1), 207 (13), 204 (17), 203 (100), 133 (33), 119 (13), 105 (11), 95 (12), 81 (10), 69 (10), 55 (11)
57.8–58.0	Betulin	442 (8), 203 (100), 189 (77), 133 (66), 121 (55), 107 (57), 105 (49), 95 (56), 93 (54), 81 (67)
Acids analyzed after methylation:		
42.0–42.2	Olean-2,12-dien-28-oic acid methyl ester	452(11), 425 (9), 263 (11), 262 (61), 221 (14), 203 (100), 190 (15), 189 (22), 133 (14), 119 (12)
44.5–44.8	Ursa-2,12-dien-28-oic acid methyl ester	452 (12), 425 (9), 263 (20), 262 (100),221 (27), 203 (79), 190 (18), 189 (27), 133 (58), 119 (23)
45.3–45.5	3-Oxo-olean-12-en-28-oic	468 (6), 262 (32), 204 (17), 203 (100), 202 (21), 189

	acid methyl ester	(29),133 (17), 119 (14), 105 (12), 55 (12)
46.0–46.6	Oleanolic acid methyl ester	470 (1), 262 (48), 207 (13), 204 (16), 203 (100), 202 (21), 189 (22), 133 (17), 119 (13), 105 (14)
46.6–47.2	Betulinic acid methyl ester	470 (5), 207 (41), 203 (38), 189 (100), 175 (40), 119 (41), 107 (38), 105 (37), 95 (37), 93 (38)
47.5–48.0	3-Oxo-urs-12-en-28-oic acid methyl ester	468 (3), 263 (21), 262 (96), 249 (20), 204 (17), 203 (100), 189 (29), 133 (79), 119 (30), 105 (19)
49.0–51.0	Ursolic acid methyl ester	470 (1), 263 (20), 262 (100), 207 (32), 203 (93), 189 (29), 133 (76), 119 (34), 105 (21), 95 (18)
60.0–61.0	Maslinic acid methyl ester	486 (2), 263 (10), 262 (53), 204 (17), 203 (100), 202 (20), 189 (20), 133 (16), 119 (13), 105 (12), 69 (10)
63.0–64.0	Corosolic acid methyl ester	486 (1), 263 (15), 262 (74), 204 (17), 203 (100), 202 (22), 189 (21), 119 (18), 105 (14), 55 (12)
73.0–74.0	Pomolic acid methyl ester	486 (3) 263 (12), 263 (55), 204 (16), 203 (100), 202 (20), 189 (17), 119 (12), 105 (10), 75 (14)

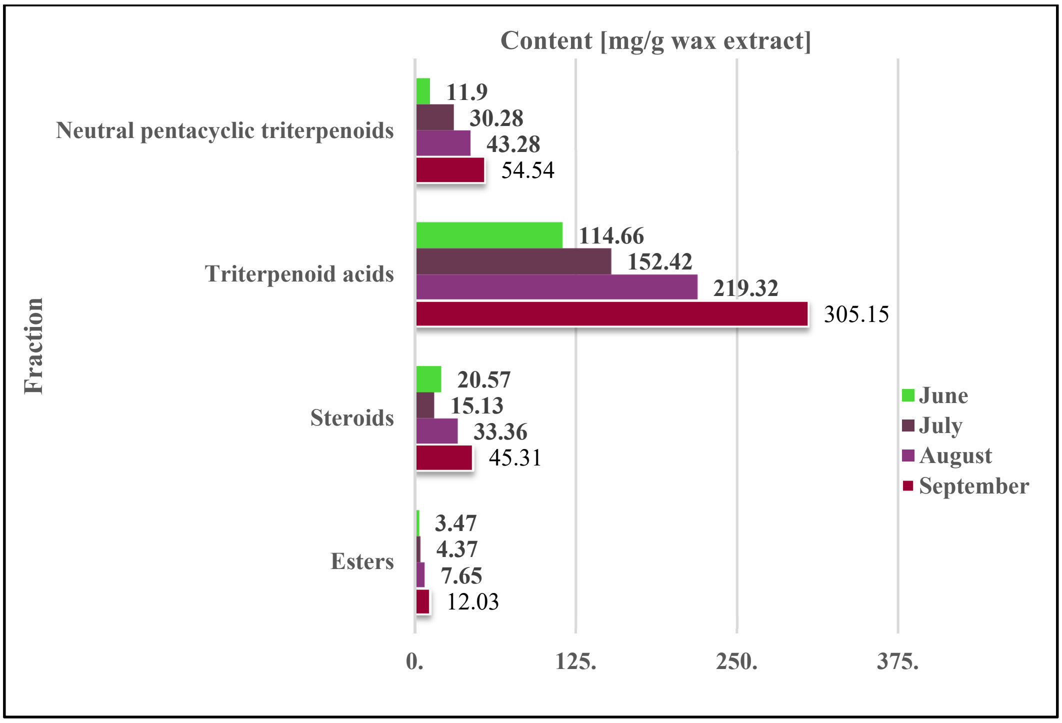

Figure S1. Changes in the content of triterpenoids in cuticular waxes during rugosa rose *Rosa rugosa* fruit development.

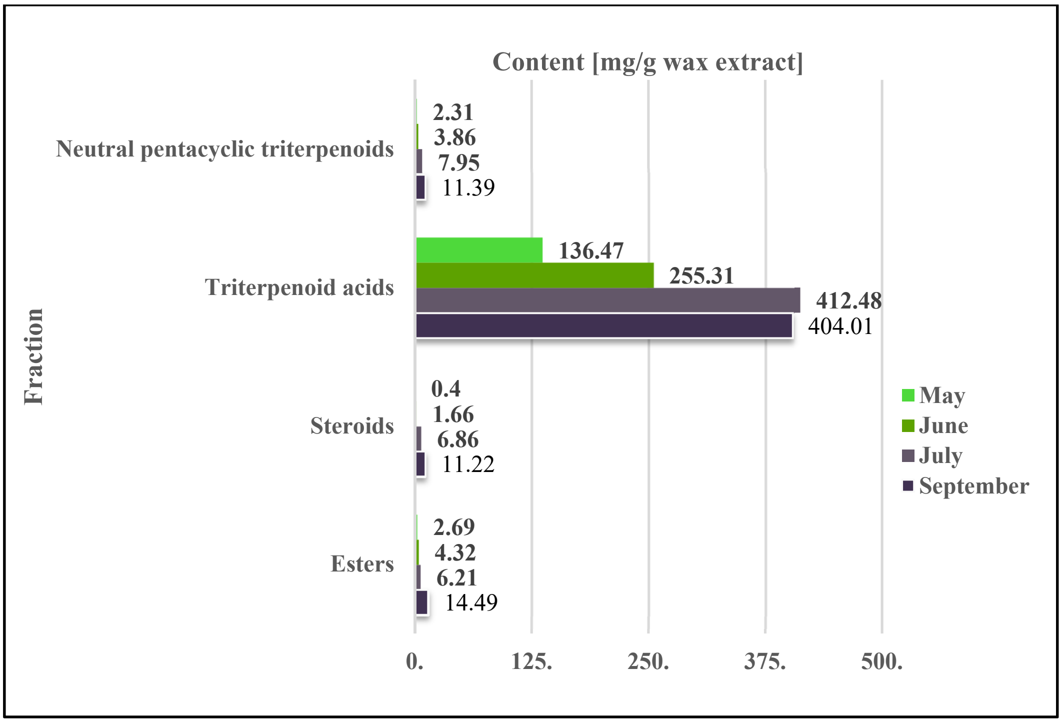

Figure S2. Changes in the content of triterpenoids in cuticular waxes during black chokeberry *Aronia melanocarpa* var. Galicjanka fruit development.

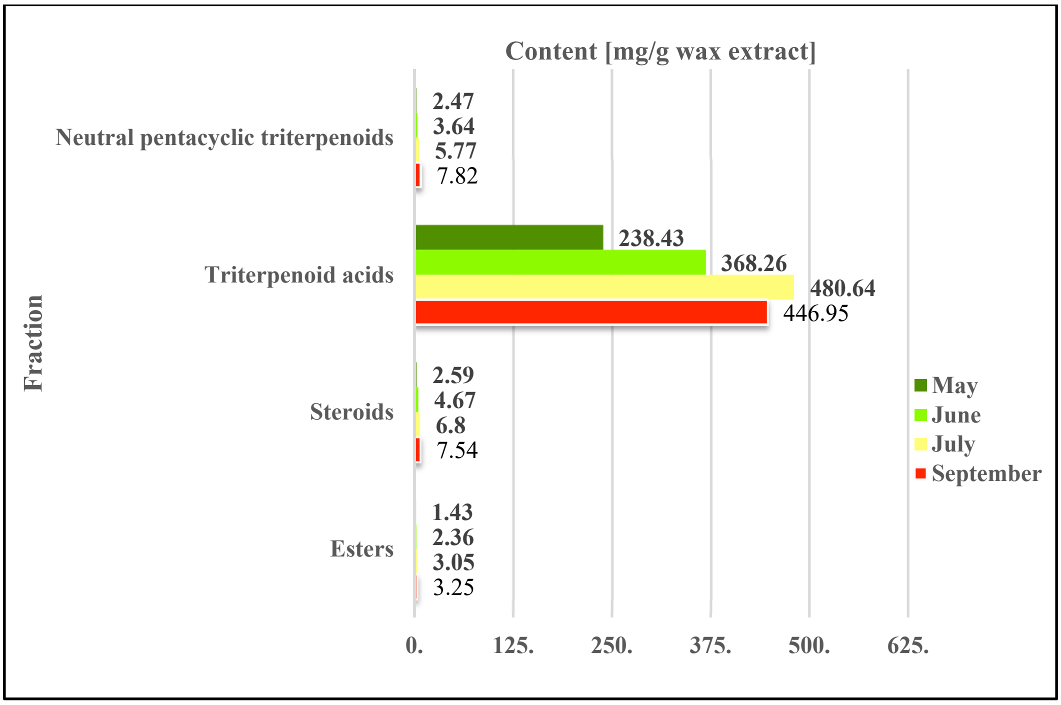

Figure S3. Changes in the content of triterpenoids in cuticular waxes during apple *Malus domestica* var. Antonovka fruit development.
